# The Dysregulation of microRNA-30b Directly Regulates Cystathionine Gamma-Lyase and Exhibits Poor Invasion Activity in Preeclampsia

**DOI:** 10.7759/cureus.78036

**Published:** 2025-01-26

**Authors:** Shobhit Saxena, Bincy A John, Neelam Verma, Manisha Mishra

**Affiliations:** 1 Anatomy, All India Institute of Medical Sciences, New Delhi, New Delhi, IND; 2 Imaging Scientist, Charles River Laboratories, Durham, USA; 3 Anatomy, Maulana Azad Medical College, New Delhi, IND; 4 Anatomy, D Y Patil School of Medicine, Mumbai, IND

**Keywords:** american congress of obstetrics and gynaecology (acog), amino acetic acid (aoaa), hydrogen sulphide (cth/cse), ischemia-reperfusion injury, matrix metalloproteases, micro-rna, preeclampsia-eclampsia, pregnancy hypertension, sodium hydrosulphide (nahs), tissue inhibitor of metalloproteinases

## Abstract

Background and objective

Preeclampsia (PE) is a severe pregnancy complication characterized by high blood pressure and proteinuria after 23 weeks of gestation. Insufficient trophoblast invasion has been linked to its development. Hydrogen sulfide (H₂S), with its genes cystathionine gamma-lyase (CTH) playing the role of an important vasodilator, may be associated with various diseases including PE. This study investigated the role of microRNA-30b (miR-30b) in trophoblast invasion using HTR-8/SVneo cells, blood, and placental samples

Methods

The study involved samples from 40 preeclamptic subjects and 40 healthy controls. Gene expression was analyzed via quantitive real-time PCR and enzyme-linked immunosorbent assay (ELISA). The effects of H₂S were simulated using a sodium hydrogen sulfide (NaHS) inhibitor (AOAA), and miR-30b inhibitors were used to assess changes in invasion capacity.

Results

NaHS treatment enhanced trophoblast invasion, and hypoxia/reoxygenation (H/R) + NaHS-treated cells showed increased invasion compared to H/R-treated cells alone. miR-30b inhibition led to higher expression of CTH, matrix metalloproteinase-9 (MMP-9), and reduced tissue inhibitor of metalloproteinases 1 (TIMP-1) and TIMP-2 expression, improving cell invasion. Patient samples showed lower CTH, MMP-2, and MMP-9 levels in PE, with elevated TIMP-1 and TIMP-2. Protein expression also revealed reduced CTH and MMP-9 in preeclamptic patients.

Conclusions

Based on our findings, miR-30b influences trophoblast invasion by modulating CTH expression and the MMP/TIMP balance. Enhanced H₂S production improves invasion, suggesting that miR-30b and related pathways can be potential therapeutic targets for PE management.

## Introduction

Preeclampsia (PE) is a multifactorial and complex pregnancy-associated hypertensive disorder [1]. Hypertensive disorders of pregnancy constitute one of the leading causes of maternal mortality worldwide during pregnancy. Various factors such as genetic, environmental, protein oxidation, DNA damage, and apoptosis affect immune cells and approximately 2-8% of all pregnancies, according to the American Congress of Obstetrics and Gynaecology (ACOG), with a significant impact on maternal and fetal health. The ACOG 2019 report has reported an annual worldwide PE-related maternal mortality of nearly 50,000-70,000 [2]. In pregnancy, after 20 weeks of gestation with clinical onset of hypertension >140/110 mmHg systolic and >90 mmHg diastolic and proteinuria >300 mg/24 hr, PE continues to be a leading cause of maternal and neonatal morbidity and mortality, according to the ACOG [3,4].

This condition is often classified into early-onset (before 34 weeks) and late-onset (after 34 weeks), with each having distinct etiologies [5]. However, the hallmark in both scenarios is the failure of trophoblast invasion into the maternal spiral arteries, leading to placental ischemia [1], which results in systemic endothelial dysfunction [6]. Central to the pathogenesis of PE is the dysregulation of hydrogen sulfide (H₂S), a gasotransmitter with potent vasodilatory, anti-inflammatory, and cytoprotective properties [7]. H₂S is primarily synthesized by cystathionine gamma-lyase (CTH), and its deficiency has been implicated in impaired placental development and trophoblast invasion [7]. The importance of trophoblast invasion lies in its role in remodeling maternal spiral arteries, a process essential for establishing an adequate uteroplacental blood flow [8,9]. However, the molecular mechanisms that control trophoblast invasion in PE remain less understood [9,10].

Recent studies have identified microRNAs (miRNAs) as critical post-transcriptional regulators of gene expression in various physiological and pathological processes, including trophoblast invasion. miRNAs play critical roles in regulating trophoblast invasion by modulating the activity of matrix metalloproteinases (MMPs), which are key enzymes involved in the degradation of the extracellular matrix (ECM) [11]. In particular, microRNA-30b (miR-30b) has emerged as a key modulator of CTH expression and, by extension, H₂S production. miR-30b directly targets the 3’-untranslated region (3’-UTR) of the CTH gene, suppressing its expression and leading to reduced H₂S levels. This regulation of H₂S has been linked to various biological processes, including tissue injury protection [12].

This reduction in H₂S subsequently impacts the activity of MMP-2 and MMP-9, which are responsible for ECM degradation and the facilitation of trophoblast invasion. Research shows that the downregulation of H₂S decreases the expression and activity of MMP-2 and MMP-9, impairing trophoblast migration and invasion [13]. Concomitantly, increased levels of tissue inhibitors of metalloproteinases (TIMP-1 and TIMP-2) further impede this process by inhibiting MMP activity. Elevated TIMP expression has been shown to inhibit MMP-2 and MMP-9 activities, contributing to reduced trophoblast invasion in conditions such as early-onset PE [14].

The imbalance between MMPs and TIMPs is well-documented in the context of early-onset PE, with several studies, including that of Arora et al., reporting reduced MMP-2 and MMP-9 levels and elevated TIMP-1 and TIMP-2 levels in preeclamptic placentas. This imbalance leads to the decreased gelatinolytic activity of MMPs, as confirmed through zymography, and contributes to impaired trophoblast invasion, a key factor in the pathophysiology of early-onset PE. Arora et al. also emphasized that the observed downregulation of MMPs and concurrent upregulation of TIMPs in early-onset PE provides a molecular basis for the impaired placental development in this condition [14]. However, the precise regulatory role of miR-30b in this dysregulation remains to be elucidated. While miR-30b has been shown to inhibit trophoblast invasion, its specific influence on MMP and TIMP regulation in PE is still under investigation [15].

In this study, we aim to explore the functional relationship between miR-30b, CTH, MMPs, and TIMPs in the context of trophoblast invasion and PE. We hypothesize that miR-30b modulates trophoblast invasion by regulating CTH and its downstream effects on MMPs and TIMPs, ultimately contributing to the impaired placentation observed in PE.

## Materials and methods

Study design and patient samples

The study involved conducting experiments in a controlled environment and analyzing samples with the aid of the Statistics Department of All India Institute of Medical Sciences (AIIMS), New Delhi. Data were analyzed using Graph Pad Prism/Stata 14 and presented as mean ± standard deviation (SD). Data following normal distribution were compared by one-way ANOVA followed by multiple comparisons using the Bonferroni test. On the other hand, data not following normal distribution were compared by the Kruskal-Wallis test and followed by Dunn's multiple comparison test with Bonferroni correction. Paired t-test and Wilcoxon rank sum test were used to compare the average level of the variable between the two groups. A p-value <0.05 was considered statistically significant.

Forty PE patients and 40 age-matched healthy controls were finally included in the study. PE cases were not subdivided into early- and late-onset types. All participants were recruited from the Department of Obstetrics and Gynaecology after obtaining ethical approval from the Institutional Ethics Committee of AIIMS, New Delhi (approval no: IEC/T-390/22.07.2015), and all participants provided informed consent. Various clinical characteristics, such as maternal age, gestational age, systolic/diastolic blood pressure, and proteinuria levels, were documented (Table 1).

**Table 1:** Clinical characteristics of the cohort SD: standard deviation

Characteristics	Study group			T score test (p-value)	t value	df value	Mean difference	95% confidence interval	R squared value
	Normotensive, nonproteinuric (controls) (n=40)	Preeclampsia (n=40)	P-value						
Maternal age, years, mean ± SD	29 ± 4.8	36.32 ± 1.32	>0.05	0.0179	2.51	29	2.133	1.10 to 0.0568	0.1785
Gestational age, weeks, mean ± SD	36.38 ± 1.35	36.32 ± 1.32	>0.05	0.2896	1.079	29	0.4333	1.255 to 0.3881	0.0386
Systolic blood pressure, mmHg, mean ± SD	121.47 ± 6.62	158.32 ± 10.95	<0.0001	0.0155	2.572	29	21.13	11.84 to 103.8	0.1857
Diastolic blood pressure, mmHg, mean ± SD	81.87 ± 3.0	104.1 ± 8.28	<0.0001	<0.0001	14.55	29	21.13	18.16 to 24.10	0.8795
Proteinuria, grams/day	Nil	3+	N/A	<0.0001	29	29	2.9	2.696 to 3.105	0.9667

Cell culture and treatments

The HTR-8/SVneo trophoblast cell line, which originates from first-trimester extravillous trophoblast transfected with simian virus 40 large T antigen (SV40) was utilized for all in vitro experiments. The cells were grown in Dulbecco's Modified Eagle Medium (DMEM) supplemented with 10% fetal bovine serum (FBS). In the experimental treatments, the cells were either exposed to 100 μM NaHS (an H₂S donor) for 10 minutes or pre-treated with 1 μM AOAA (CTH inhibitor) for 24 hours. To simulate ischemia-reperfusion injury, the cells underwent hypoxia/reoxygenation (H/R) cycles, followed by recovery with or without NaHS supplementation.

Transwell Invasion Assay

Trophoblast invasion was assessed using a Matrigel-coated transwell invasion assay. HTR-8/SVneo cells (1 × 10⁵) were seeded in serum-free medium on Matrigel-coated filters. Cells were treated with NaHS, AOAA, H/R, or a combination of H/R + NaHS. After 24 hours, non-invading cells were removed, and the invaded cells were fixed, stained with crystal violet, and counted under a microscope in five random fields. Results were expressed as percentage invasion relative to control (Tables 2-4).

**Table 2:** Hypoxia standardization procedure H/R: hypoxia/reoxygenation

Time of exposure to cells		Status of cell invasion	
Hypoxia (H) 5% (O_2_, 5% CO_2_, 90% N_2_)	Reoxygenation (R) (20% O_2_, 5% CO_2_ and atmospheric air)	One cycle H/R	Two cycle H/R
1 hour	1 hour	Cell invasion seen	Cell invasion seen
2 hour	1 hour	Cell invasion seen	Cell invasion delayed
4 hours	1 hour	Cell invasion delayed	Cell invasion delayed

**Table 3:** AOAA dose standrization in HTR8/Svneo cells

AOAA dose	Morphology of HTR-8/SVneo cells after different time periods of AOAA treatment		
	5 minutes	10 minutes	20 minutes
25 mM	No changes in cell morphology	No changes in cell morphology	No changes in cell morphology
50 mM	No changes in cell morphology		No changes in cell morphology
100 mM	Few cells showed altered morphology	Few cells showed altered morphology	Reduced proliferation and altered morphology seen in many cells
150 mM	The number of dead cells increased	The number of dead cells increased	Number of dead cells increased

**Table 4:** NaHS standrization over HTR8/SVneo cells NaHS: sodium hydrogen sulfide

NaHS dose	Morphology of HTR-8/SVneo cells after different time periods of NaHS treatment		
	5 minutes	10 minutes	20 minutes
25 mM	No changes in cell morphology	No changes in cell morphology	No changes in cell morphology
50 mM	Very few cells invaded; no difference with controls	Few cells, no difference with controls	Cells had clear morphology; floating dead cells were also seen
100 mM	Few cells invaded	Distinct morphology, clear morphology	More dead cells were seen floating around
150 mM	Cells clear; dead cells were floating	Floating dead cells were seen	More dead cells were seen floating around

Transfection With microRNA-30b Mimic and Inhibitor

To modulate miR-30b expression, HTR-8/SVneo cells were transfected with either a miR-30b mimic or an inhibitor using Lipofectamine RNAiMAX (Thermo Fisher Scientific, Waltham, MA), following the manufacturer’s protocol (Table 5). Transfection efficiency was confirmed via qRT-PCR.

**Table 5:** Primer sequence of microRNA 30b, universal primer, and negative control and positive control RNU6

Name	Primer sequence
Negative control	ACCAAGUUUCAGUUCAUGUAAACAUCCUACACUC AGCUGUAAUACAUGGAUUGGCUGGGAGGUGGAUGU UUACUUCAGCUGACUUGGA
Universal primer	Provided by Thermo
>hsa-miR-30b-3p MIMAT0004589	CUGGGAGGUGGAUGUUUACUUC
>hsa-mir-30b MI0000441	ACCAAGUUUCAGUUCAUGUAAACAUCCUACACUCA GCUGUAAUACAUGGAUUG GCUGGGAGGUGGAUGUUUACUUCAGCUGACUUGGA
RNU6	Provided by Qiagen, Germany

Quantitative Real-Time PCR (qRT-PCR)

RNA was extracted from HTR-8/SVneo cells, patient plasma, and placental tissues using the TRIzol reagent. To generate cDNA, the RevertAid First Strand cDNA Synthesis Kit (Thermo Fisher) was utilized. SYBR Green PCR master mix was used for real-time PCR on a CFX96 Touch Real-Time PCR Detection System (Table 6). The target genes included CTH, MMP-2, MMP-9, TIMP-1, and TIMP-2, with GAPDH serving as the internal control. Relative gene expression was calculated using the 2-ΔΔCt method (Livak method). 

**Table 6:** Primers sequence used for qRT-PCR qRT-PCR: quantitative real-time polymerase chain reaction

Name	Primer sequence
CTH F R	CCCCACAAACCCCACCCAGAA GACACCAGGCCCATTAC
MMP2 F R	TCCGTGTCCTGTAAATCTGCT GACCTGAACCATAACGCACA
MMP9 F R	TTCAACGGTCGGGAATACAG AGCCATACTTGCCATCCTTC
TIMP1 F R	CCTTTGCATCTCTGGCATCT GGGAACCCATGAATTTAGCC
TIMP2 F R	CTCGCTGGACGTTGGAGGAAAGAA GTCATCTTGATCTCATAACGCTGG
GAPDH F R	TCCTGTTCGACAGTCAGCCGCA GCGCCCAATACGACCAAATCCGT

Enzyme-Linked Immunosorbent Assay (ELISA)

Protein levels in patient plasma for CSE, MMP-2, MMP-9, TIMP-1, and TIMP-2 were assessed using ELISA kits obtained from Abcam (Abcam, Cambridge, UK). The plates underwent scanning at 450 nm on a microplate reader, and concentrations were determined with reference to a standard curve.

Statistical analysis

All experiments were conducted in triplicate. The study was conducted using GraphPad Prism version 10.0.0 for Windows (GraphPad/Stata Software, Boston, MA). ANOVA was utilized to compare multiple groups, while Student’s t-test was employed for comparisons between the two groups. All p-values <0.05 were considered statistically significant.

## Results

Hydrogen sulfide enhances trophoblast invasion

**Figure 1:**
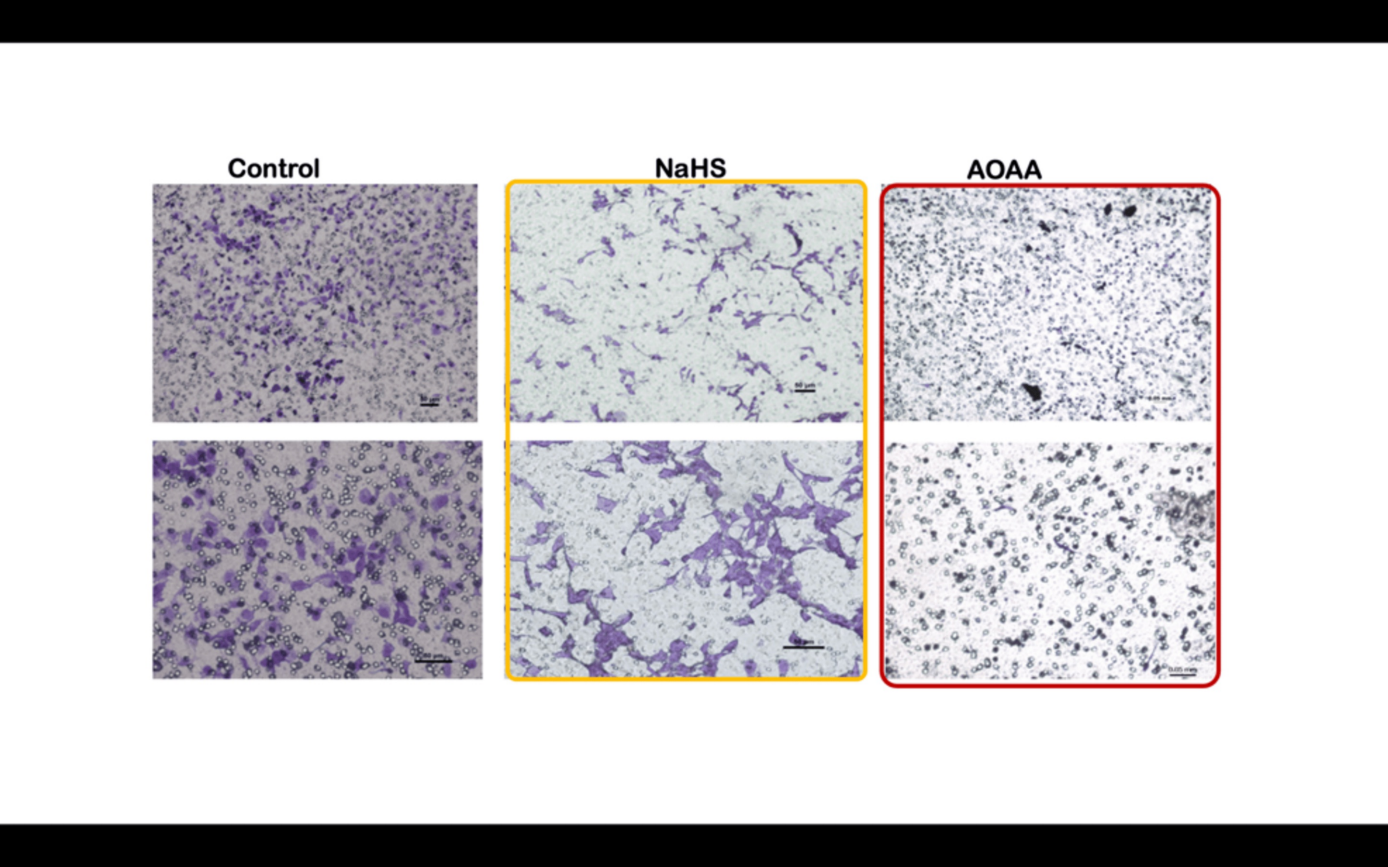
Number of invaded cells increased after various treatments The number of invaded cells increased after NaHS treatment while a decline was seen after AOAA treatment. Representative photomicrographs showing the HTR-8/SVneo cells invaded on the under surface of transwell inserts at different magnifications. A1, A2: Group 1 (controls) at different magnifications; B1, B2: Group 2 (NaHS) at different magnifications; C1, C2: Group 3 (AOAA). The percentage invasion was significantly increased in NaHS-treated cells as compared to untreated cells (controls) whereas inhibitor of H_2_S-producing enzyme (i.e. AOAA) showed a significantly reduced percentage of invaded cells. Scale bar: 50 µm H_2_S: hydrogen sulfide; NaHS: sodium hydrogen sulfide

The capacity of trophoblast cells to invade the ECM is a critical process in the establishment of successful placentation. Our initial experiments demonstrated the profound impact of H₂S on trophoblast invasion. NaHS, an H₂S donor, significantly increased the invasion capacity of HTR-8/SVneo cells, as evidenced by a marked increase in both the number of invaded cells and the percentage of invasion. Invasion assay results showed that when exposed to 100 μM NaHS, the number of cells that penetrated the Matrigel barrier increased by over 93% compared to control cells (p<0.0001) (Figure 1A). The percentage of invasion in NaHS-treated cells was 48.05 ± 8.19%, compared to the control group’s 24.80 ± 2.35% (p<0.0001). The invasion index, a normalized measure of invasion, was 1.93 ± 0.27 in NaHS-treated cells compared to 1.00 in controls (p<0.0001). This result highlights the significant pro-invasive role of H₂S in trophoblast cells.

In contrast, cells treated with AOAA, a known inhibitor of CSE, showed a drastic reduction in their invasion capacity. The number of invaded cells dropped by over 65% compared to controls (p<0.0001), with a corresponding decrease in the percentage invasion (8.6 ± 4.57%, p<0.0001) and an invasion index of 0.34 ± 0.17 (p<0.0001) (Figure 1B). These results confirm the role of CSE, and by extension, H₂S, as a positive regulator of trophoblast invasion.

Protective role of H₂S in hypoxia/reoxygenation (H/R)-induced injury

To mimic the ischemic conditions often present in preeclamptic placentas, HTR-8/SVneo cells were subjected to H/R cycles, a model designed to simulate the intermittent oxygenation changes observed in placental ischemia. H/R exposure significantly decreased trophoblast invasion, with 1200 ± 56.14 cells invading the Matrigel barrier compared to 2480 ± 235.52 cells in the control group (p<0.0001) (Figure 1C). The percentage invasion in H/R-treated cells was 12 ± 3.40%, and the invasion index was 0.48 ± 0.13, both significantly reduced compared to controls (p<0.0001).

Interestingly, NaHS supplementation following H/R injury partially restored invasion capacity. The number of invaded cells increased to 3754 ± 888.11 (p<0.0001), with a corresponding increase in percentage invasion to 37.54 ± 8.8% (p<0.001) and invasion index to 1.54 ± 0.48 (p<0.001) (Figure 1D). These findings suggest that H₂S plays a cytoprotective role in trophoblast cells exposed to ischemia-reperfusion injury, potentially through the upregulation of matrix-degrading enzymes and the restoration of cellular functions.

Invasion capacity after microRNA-30b treatment 

**Figure 2:**
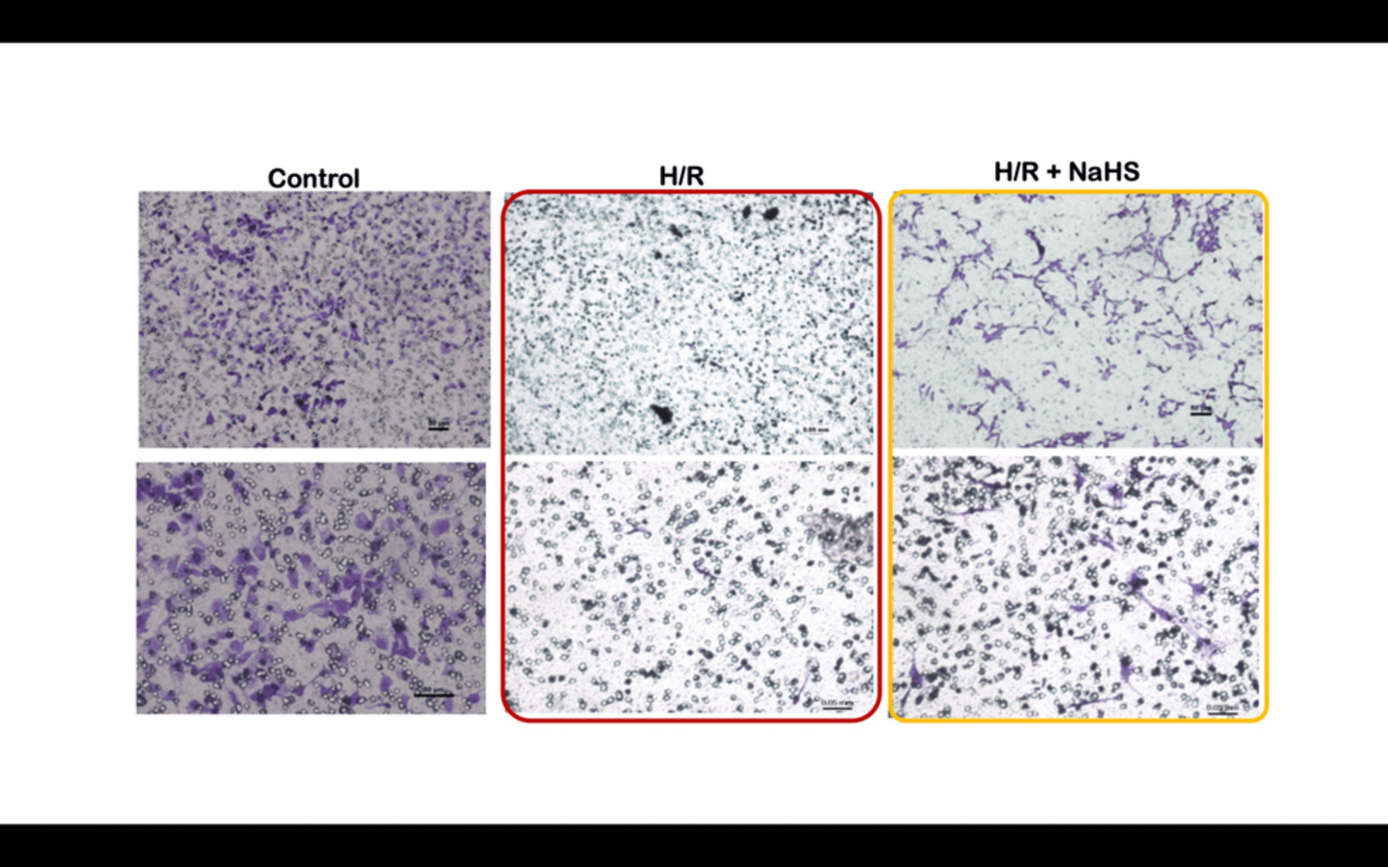
Number of invaded cells decreased on H/R treatment while it increased as NaHS supplementation was done Representative photomicrographs showing the HTR-8/SVneo cells invaded on the under surface of transwell inserts at different magnifications. A1, A2: Group 1 (controls), B1, B2: Group 4 (H/R), C1, C2: Group 5 (H/R + NaHS). The percentage invasion was significantly reduced when the cells were exposed to hypoxia-reoxygenation cycles as compared to controls. This decrease in the percentage of invasion cells improved significantly when NaHS was given to H/R-exposed cells. Scale bar: 50 µm H/R: hypoxia/reoxygenation; NaHS: sodium hydrogen sulfide

Invasion results following miR-30b modulation indicated that miR-30b mimic-transfected cells showed a significant reduction in invasion capacity, with 512 ± 81.57 cells invading compared to 1726 ± 116.38 cells in miR-30b inhibitor-transfected cells (p<0.0001). The percentage of invasion in miR-30b mimic-transfected cells was reduced to 5.12 ± 0.81%, while the invasion index dropped to 0.67 ± 0.29 (p<0.0001) (Figure 2B). In contrast, cells transfected with the miR-30b inhibitor exhibited enhanced invasion, with the percentage invasion increasing to 17.26 ± 1.16%, and the invasion index reaching 2.23 ± 0.56 (p<0.0001) (Figure 2C).

These results demonstrate that miR-30b exerts a negative regulatory effect on trophoblast invasion, primarily by suppressing CSE and H₂S production. These findings indicate that miR-30b orchestrates a complex regulatory network where the downregulation of MMPs and the upregulation of TIMPs collectively impair trophoblast invasion.

mRNA expression of preeclamptic patients of plasma and placenta 

**Figure 3:**
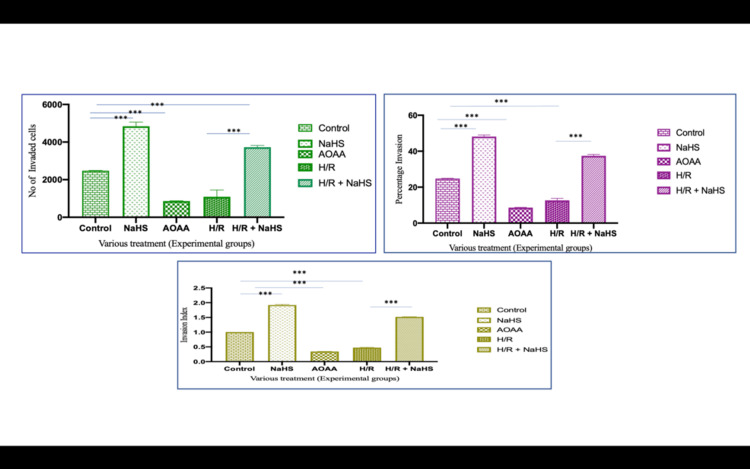
Number of invaded cells, percentage of invasion, and invasion index *P<0.05. **P<0.01. ***P<0.001 Bar diagrams represent the number of invaded cells, the percentage of invasion, and the invasion index on under surface of transwell inserts among the various experimental groups. Group 1: controls, Group 2: NaHS, Group 3: AOAA, Group 4: H/R, Group 5: H/R + NaHS. Results are representative of three independent experiments done in triplicate (n=9). On each transwell insert, 16 fields of view were used to count the number of cells H/R: hypoxia/reoxygenation; NaHS: sodium hydrogen sulfide

**Figure 4:**
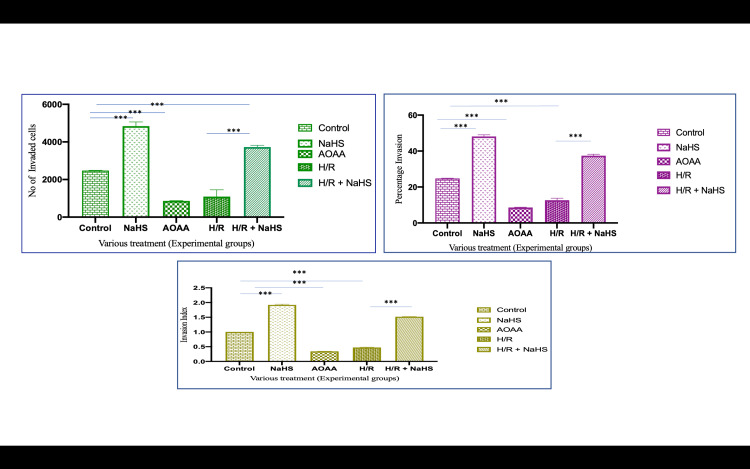
Expression of various genes after various treatments *P<0.05. **P<0.01. ***P<0.001 Bar diagrams represent the relative fold change in CTH (CSE), MMP-2, and MMP-9 mRNA levels among the various experimental groups. Group 1: controls, Group 2: NaHS, Group 3: AOAA, Group 4: H/R, Group 5: H/R + NaHS. Results are expressed as mean ± SEM. Results are representative of three independent experiments done in triplicate (n=9) CTH: cystathionine gamma-lyase; H/R: hypoxia/reoxygenation; MMP: matrix metalloproteinase; NaHS: sodium hydrogen sulfide; SEM: standard error of the mean

**Figure 5:**
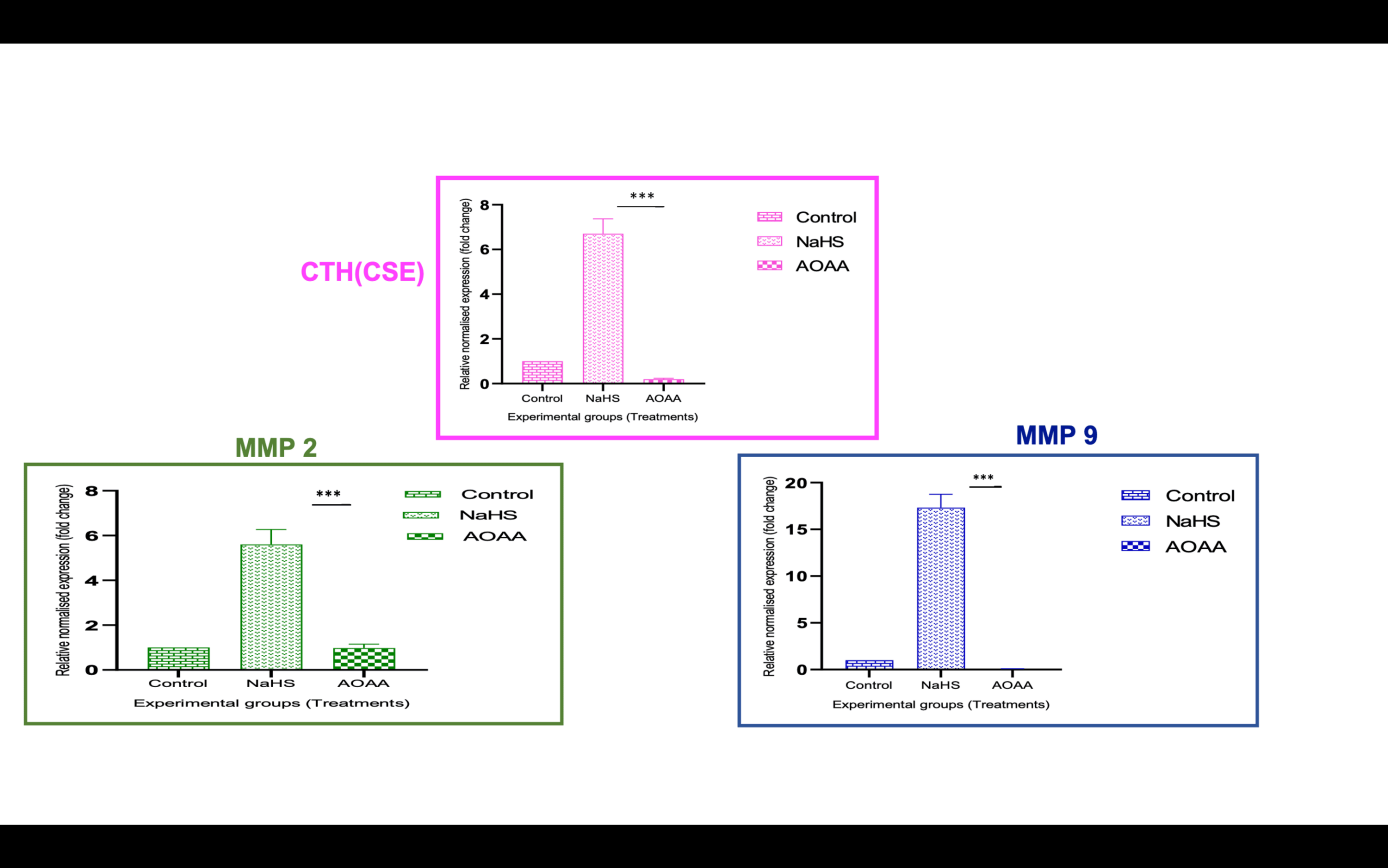
mRNA expression of CTH, MMP-2, and MMP-9 of various treatments *P<0.05. **P<0.01. ***P<0.001 CTH: cystathionine gamma-lyase; MMP: matrix metalloproteinase; NaHS: sodium hydrogen sulfide

**Figure 6:**
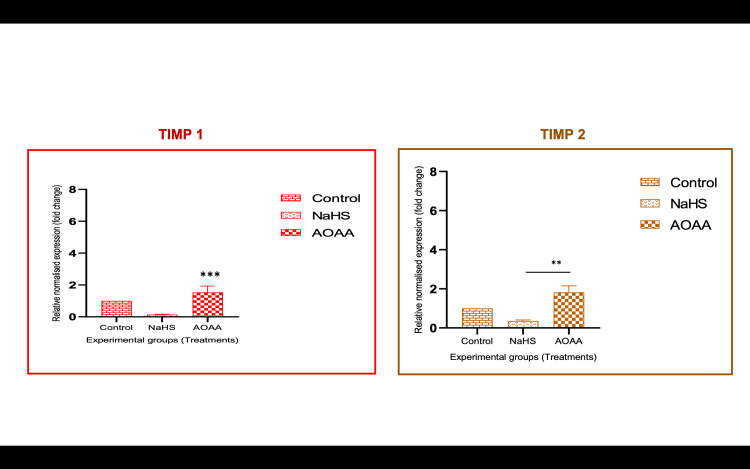
TIMP-1 and TIMP-2 mRNA expression after NaHS and AOAA treatments *P<0.05. **P<0.01. ***P<0.001 Bar diagrams represent the relative fold change in TIMP-1 and TIMP-2 mRNA levels among the various experimental groups. Group 1: controls, Group 2: NaHS, Group 3: AOAA, Group 4: H/R, Group 5: H/R + NaHS. Results are expressed as mean ± SEM. Results are representative of three independent experiments done in triplicate (n=9) H/R: hypoxia/reoxygenation; NaHS: sodium hydrogen sulfide; SEM: standard error of the mean; TIMP: tissue inhibitor of metalloproteinases

**Figure 7:**
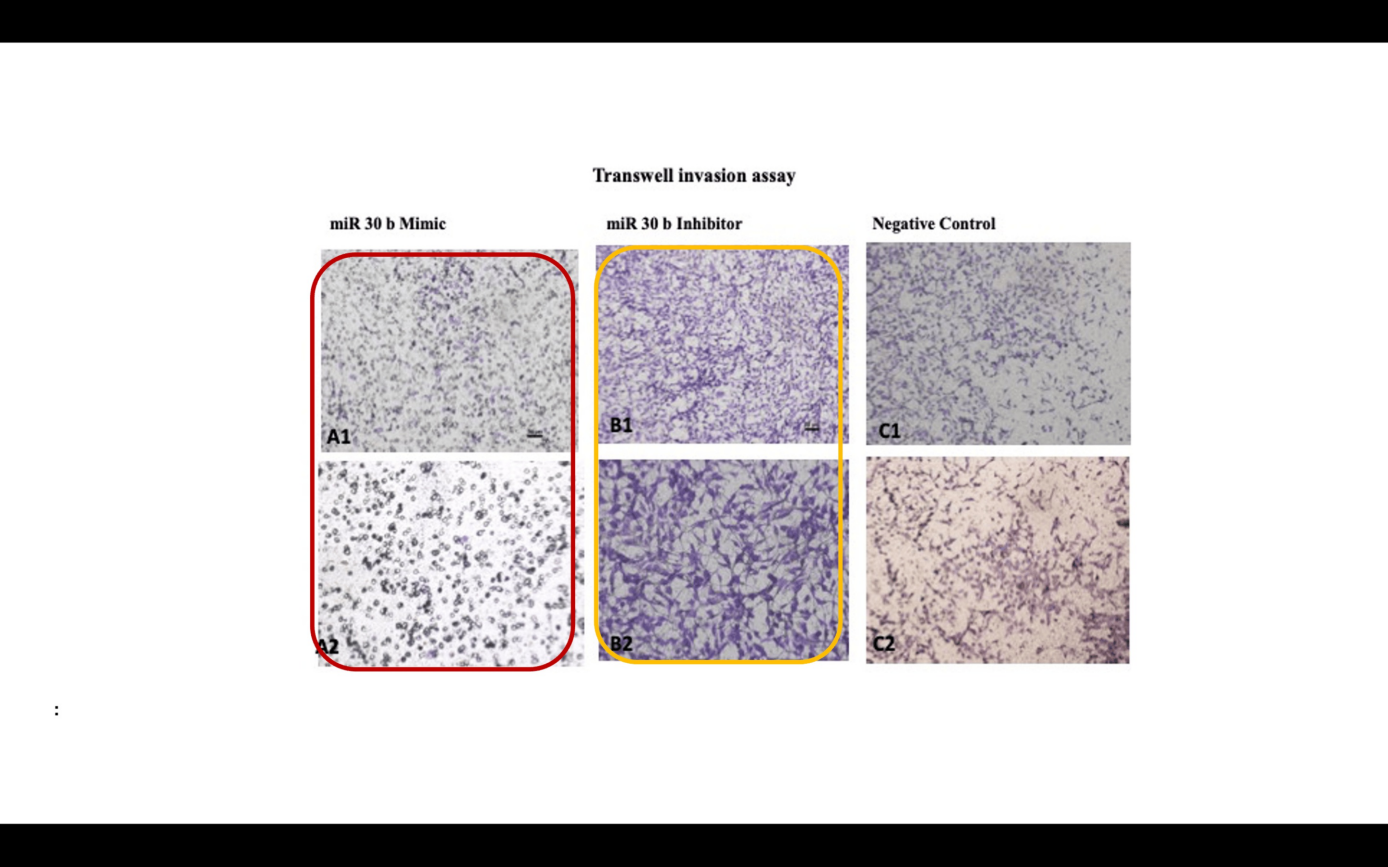
miR-30b mimic-transfected cells showed decrease in invasion whereas miR-30b inhibitor-transfected cells showed increased invasive capacity Representative photomicrographs showing the HTR-8/SVneo cells invaded on the under surface of transwell inserts at different magnifications. A1, A2: Group 6 (miR-30b mimic); B1, B2: Group 7 (miR-30b inhibitor); C1, C2: Group 8 (negative controls: RNA). The percentage of invasion significantly reduced when the cells were exposed to miR-30b mimic as compared to the inhibitor. Scale bar: 50 µm miR-30b: microRNA-30b

**Figure 8:**
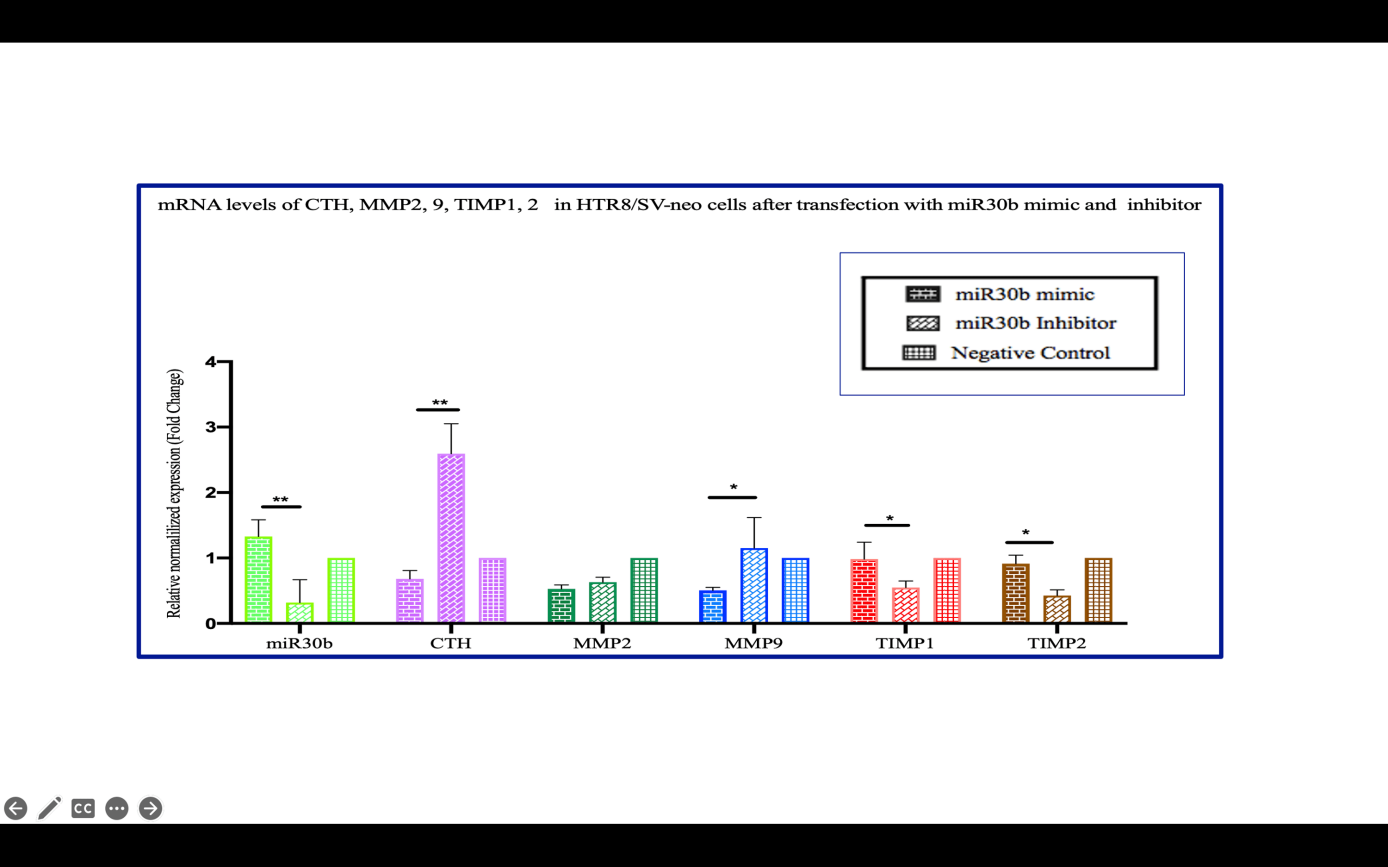
miR-30b mimic-transfected cells showed downregulation of CTH expression whereas miR-30b inhibitor upregulated CTH expression *P<0.05. **P<0.01. ***P<0.001 Bar diagrams represent the relative fold change in miR-30b, CTH (CSE), MMP-2, MMP-9, TIMP-1, and TIMP-2 mRNA levels among the various experimental groups. (Groups 6-8) (Group 6: miR-30 mimic, Group 7: miR-30 inhibitor, and Group 8: negative controls). Results are representative of three independent experiments done in triplicate (n=9) CTH: cystathionine gamma-lyase; miR-30b: microRNA-30b; MMP: matrix metalloproteinase; TIMP: tissue inhibitor of metalloproteinases

**Figure 9:**
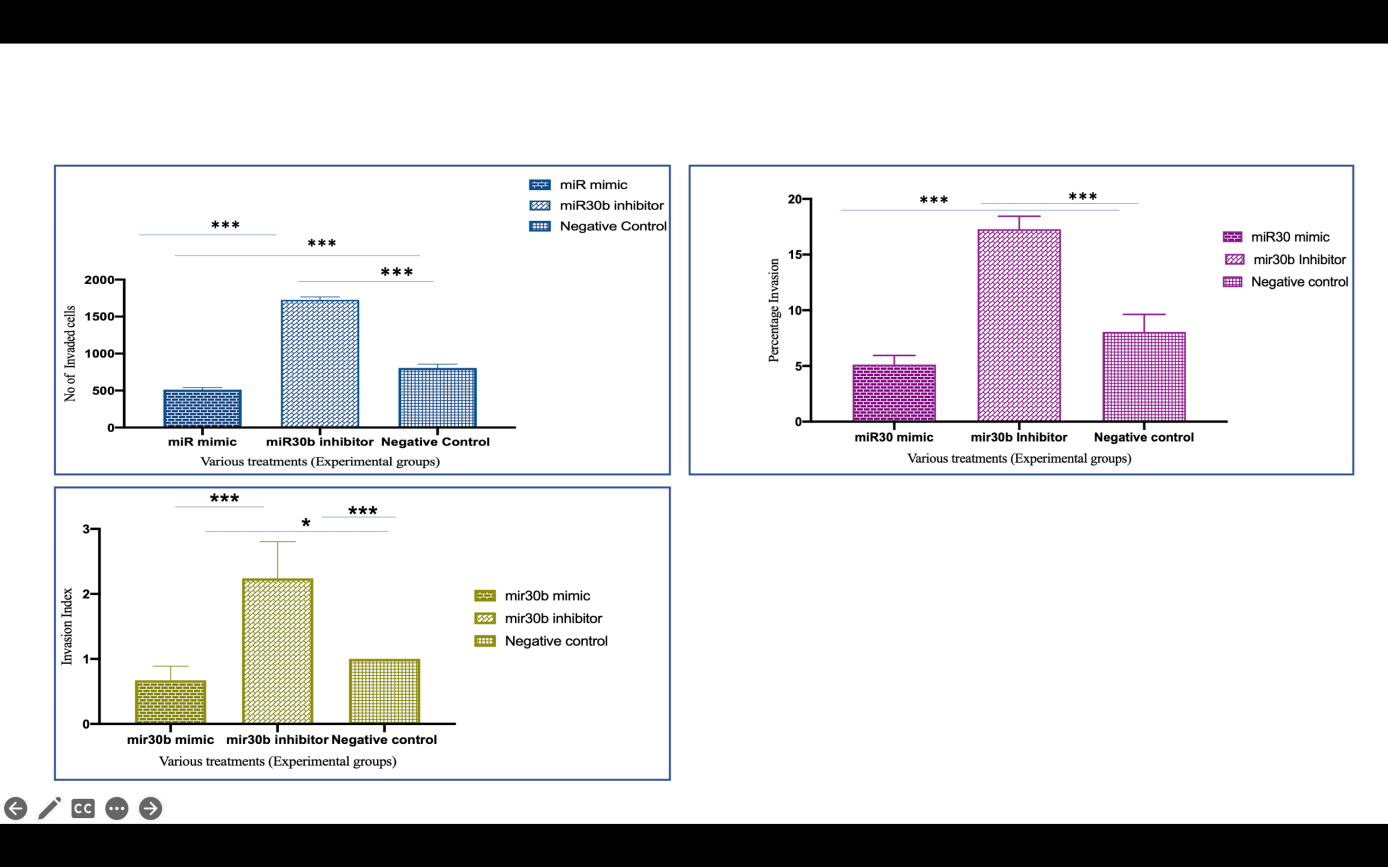
Number of invaded cells, percentage of and invasion index of trophoblast cells after miR-30b mimic and miR-30b inhibitor transfection The number of invaded cells, percentage of invasion, and invasion index increased after miR-30b inhibitor transfection, and reduction was observed after miR-30b mimic transfection miR-30b: microRNA-30b

**Figure 10:**
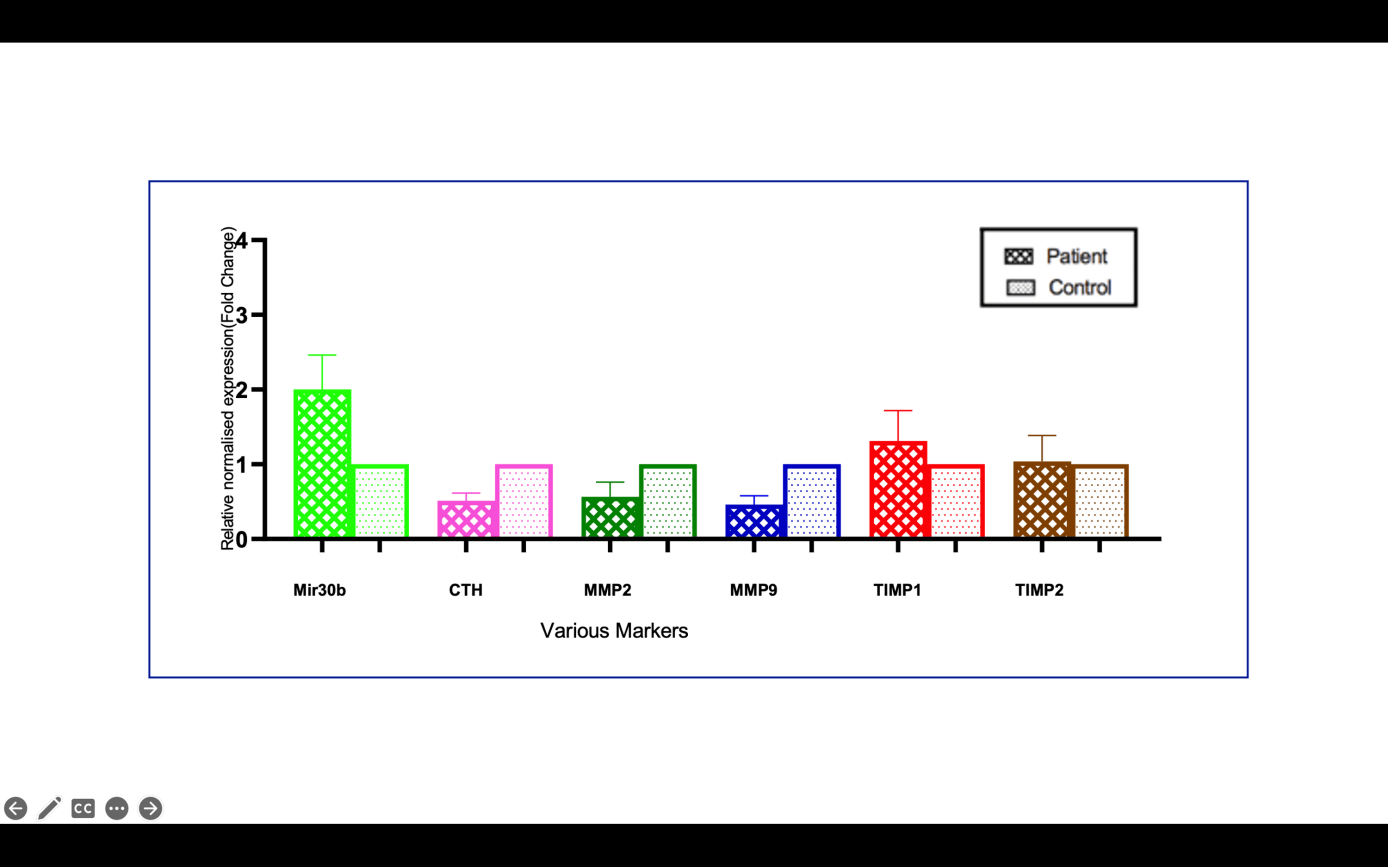
mRNA expression in the plasma of preeclampsia patients vs. controls Bar diagrams represent the relative fold change in miR-30b, CTH (CSE), MMP-2, MMP-9 TIMP-1, and TIMP-22 mRNA levels in plasma in patients and controls CTH: cystathionine gamma-lyase; miR-30b: microRNA-30b; MMP: matrix metalloproteinase; TIMP: tissue inhibitor of metalloproteinases

**Figure 11:**
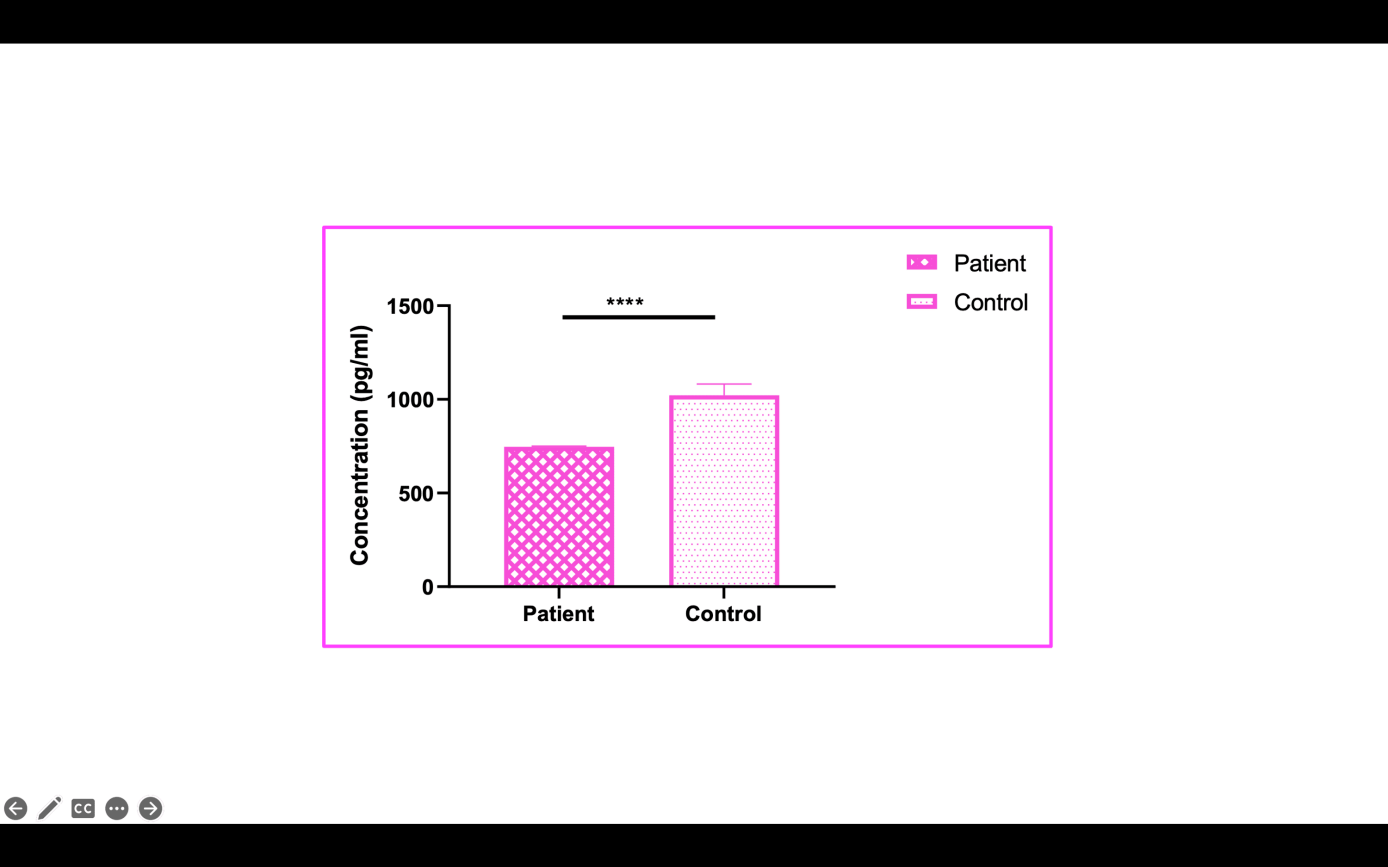
CTH levels in preeclampsia patients vs. controls CTH: cystathionine gamma-lyase

**Figure 12:**
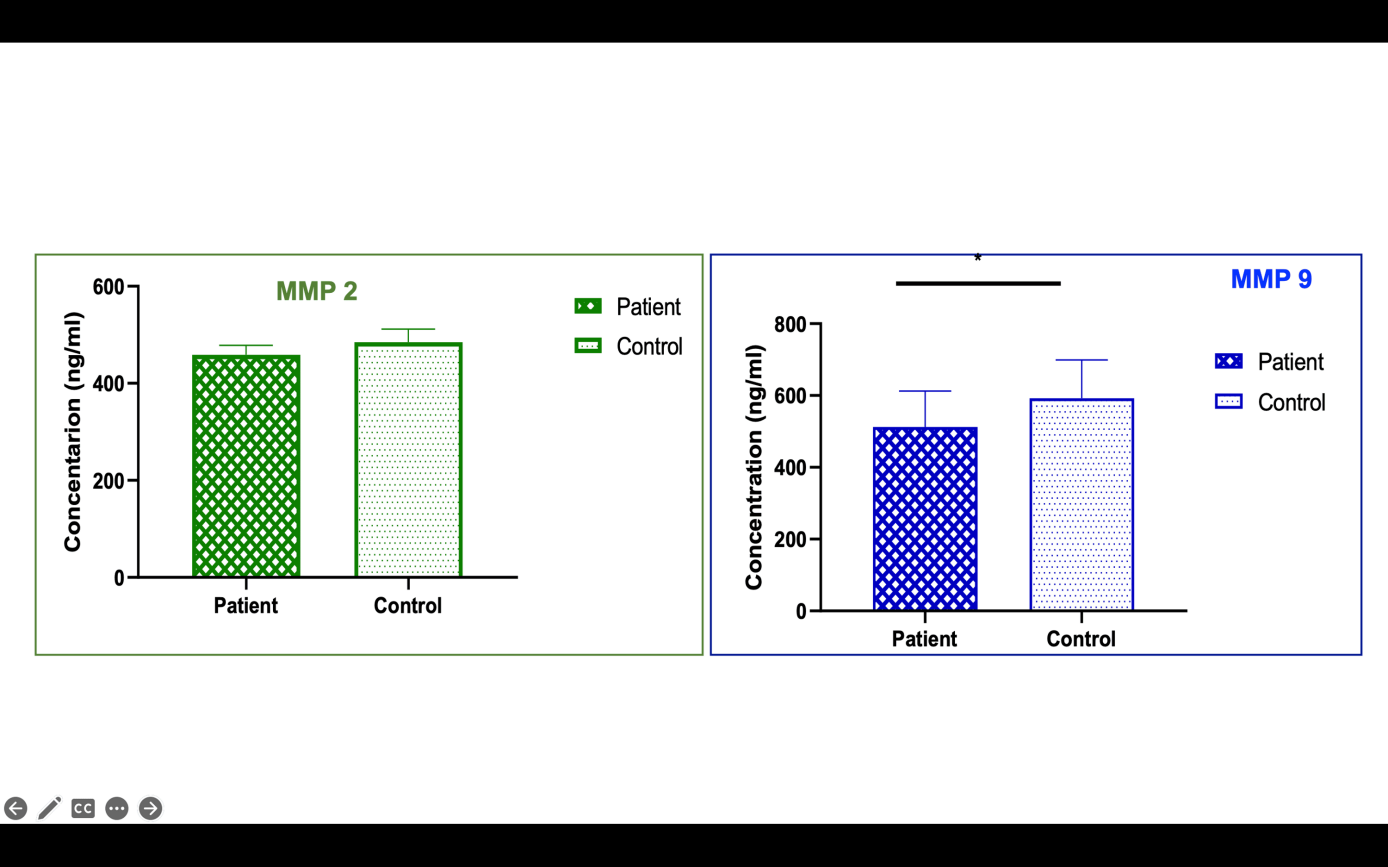
MMP-9 levels were reduced significantly in preeclamptic patients Bar diagrams represent the protein levels of MMP-2 and MMP-9 in plasma in patients and controls MMP: matrix metalloproteinase

**Figure 13:**
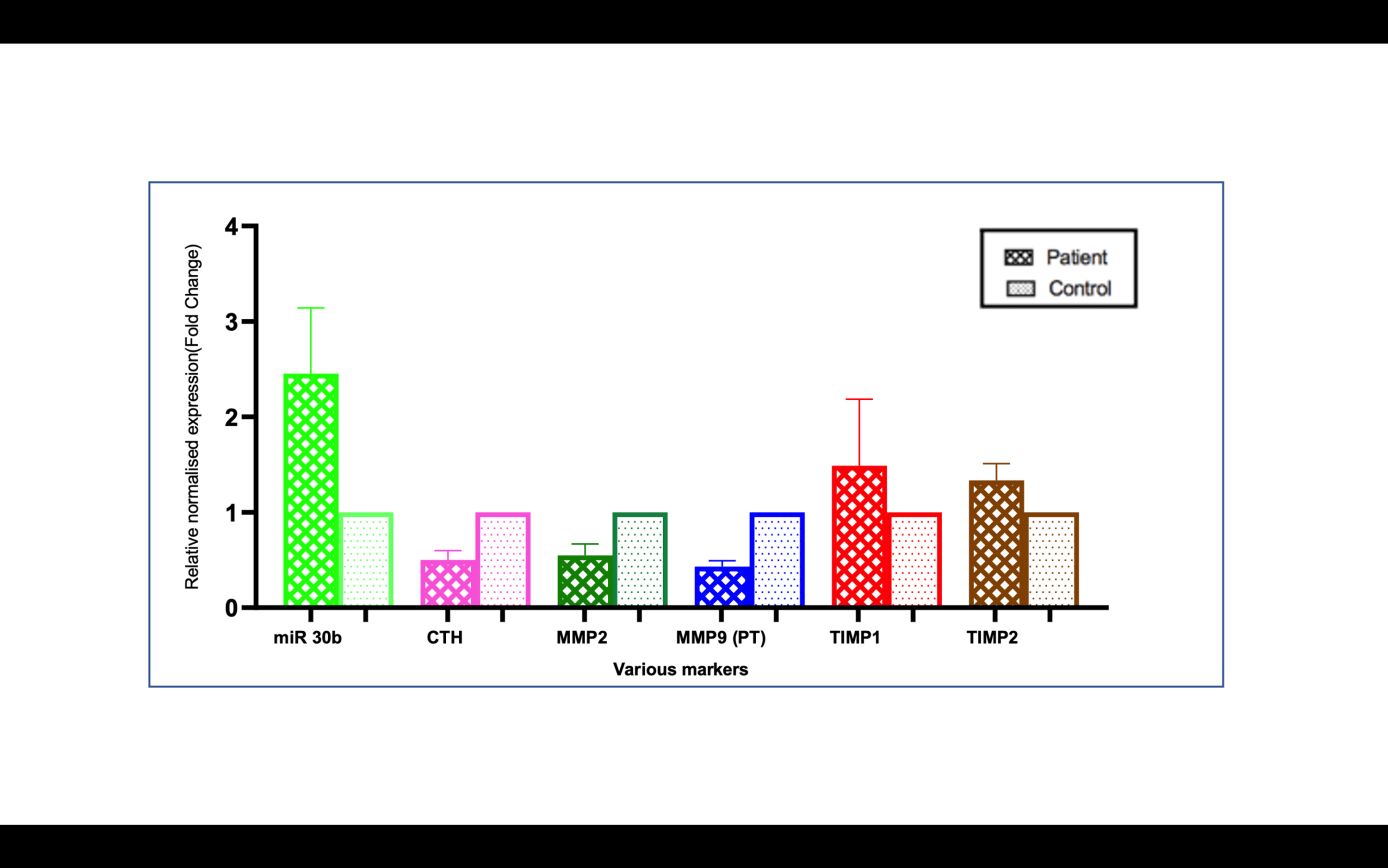
miR-30b-3p expression was upregulated in preeclamptic placenta Bar diagrams represent the relative fold change in miR-30b, CTH (CSE), MMP-2, MMP-9 TIMP-1, and TIMP-2 mRNA levels in placenta in patients and controls CTH: cystathionine gamma-lyase; miR-30b: microRNA-30b; MMP: matrix metalloproteinase; TIMP: tissue inhibitor of metalloproteinases

In the clinical arm of the study, plasma and placental samples from 40 preeclamptic patients and 40 normotensive controls were analyzed for CSE, MMP, and TIMP expression. The results mirrored those observed in the in vitro studies. CSE mRNA expression was reduced by 2.85-fold in preeclamptic plasma and by 2.0-fold in preeclamptic placentas compared to controls (p<0.001) (Figures 3, 4A). MMP-2 and MMP-9 levels were downregulated by 1.56-fold and 2.27-fold, respectively, in preeclamptic plasma, and by 1.85-fold and 2.3-fold in placentas (p<0.001) (Figure 4B). Additionally, TIMP-1 and TIMP-2 were significantly upregulated in preeclamptic plasma and placentas (p<0.001), with TIMP-1 increasing by 1.51-fold in plasma and 1.48-fold in placentas. ELISA levels were analyzed, and we found CSE levels lowered in patients compared to controls. The other proteins MMP-2, MMP-9, TIMP-1, and TIMP-2 were also analyzed. There was no difference between MMP-2 protein levels, whereas MMP-9 showed a significantly lower level in patients than controls (Figures 5-13).

These results suggest a clear molecular signature of reduced CSE/H₂S production and an imbalance in MMP/TIMP levels in preeclamptic patients, consistent with the proposed model of miR-30b-mediated regulation. The findings provide compelling evidence that microRNA-30b is a critical regulator of trophoblast invasion in PE, acting through its suppression of CSE and the subsequent reduction of H₂S levels. This regulatory pathway extends to the modulation of MMP-2, MMP-9, and their inhibitors TIMP-1 and TIMP-2, leading to impaired extracellular matrix degradation and diminished trophoblast invasion.

The data from both in vitro experiments and clinical samples converge to highlight miR-30b as a central player in the molecular pathology of PE, opening new avenues for potential therapeutic interventions targeting the CSE/H₂S pathway.

## Discussion

Role of hydrogen sulfide (H₂S) in trophoblast invasion

Our study showed the important role of H₂S in boosting trophoblast invasion by controlling CTH and downstream matrix MMP activity. This discovery is consistent with previous research that has identified H₂S as a powerful vasodilator and protective agent, especially in the context of vascular restructuring and ischemia-reperfusion injury.

Chen et al. emphasized that H₂S has significant anti-apoptotic and pro-angiogenic effects in vascular endothelial cells, which also appears to apply to placental cells [16]. However, Lu et al. provided the first molecular evidence that the increased umbilical artery resistance seen in intrauterine growth restriction (IUGR) pregnancies is associated with the remodeling of stem villus arteries, characterized by pathological smooth muscle dedifferentiation and reduced hydrogen sulfide signaling. They further showed that oxidative stress, as occurs in poor placentation, can induce SVA (stem villus arteries) remodeling, acting principally via reducing hydrogen sulfide signaling.

Our results support these findings, as treatment with NaHS not only increased trophoblast invasion but also partially restored invasion capacity after H/R injury. This protective effect of H₂S during H/R cycles reflects similar findings in non-pregnancy-related tissues, such as cardiac and renal models. For instance, Wang et al. (2012) showed that H₂S improves myocardial ischemia-reperfusion injury by reducing excessive endoplasmic reticulum stress and apoptosis [17].

Interestingly, while most studies, including those by Wang et al., emphasize the systemic vasodilatory effects of H₂S, our study highlights its critical role in local placental remodeling, specifically in the context of trophoblast invasion. This local effect is particularly significant in PE, where inadequate invasion leads to defective spiral artery remodeling, contributing to the systemic hypertensive state observed in these patients. The ability of H₂S to promote MMP-2 and MMP-9 expression in trophoblast cells offers a mechanistic explanation for its pro-invasive properties, correlating with its broader vascular roles [13,18].

miRNA-30b as a regulator of CTH and invasion

One of the novel contributions of our study is the identification of miR-30b as a key regulator of CTH expression, and by extension, H₂S biosynthesis. We found that miR-30b mimic significantly reduced CTH expression and decreased trophoblast invasion, while miR-30b inhibitor had the opposite effect, enhancing both CTH levels and invasion capacity. This suggests that miR-30b acts as a molecular brake on trophoblast invasion by downregulating CTH, leading to reduced H₂S production.

This regulatory mechanism has not been previously explored in the context of preeclampsia, although the role of miR-30 family members in other tissues is well-documented. Shen et al. 2015 demonstrated that miR-30 family inhibition protects cardiomyocytes from ischemic injury by upregulating CTH expression and restoring H₂S levels [12]. Our study extends these findings to the placenta, showing that a similar mechanism may operate in trophoblasts to regulate invasion. Moreover, while miR-30b has been shown to regulate autophagy and apoptosis in cardiac cells [17], its role in ECM remodeling via MMPs is novel to our study.

Wang et al. conducted studies that looked at the broader role of miRNAs in PE, specifically in the regulation of angiogenesis and trophoblast differentiation [19]. Our study has demonstrated the direct targeting of CTH by miR-30b, adding a new layer of understanding to the H₂S-dependent regulation of placental function. This discovery not only contributes to the increasing evidence linking miRNAs to placental pathology but also identifies miR-30b as a potential target for therapy. The restoration of trophoblast invasion in vitro by a miR-30b inhibitor suggests that inhibiting miR-30b could be a therapeutic approach to improve trophoblast function in preeclamptic pregnancies.

MMPs and TIMPs in PE

Our study confirms the dysregulation of MMPs and their inhibitors, TIMPs in PE. We observed significant downregulation of MMP-2 and MMP-9 in preeclamptic plasma and placental samples, accompanied by upregulation of TIMP-1 and TIMP-2. This imbalance between proteases and inhibitors is well-known to impair trophoblast invasion, leading to insufficient remodeling of the maternal spiral arteries and subsequent placental hypoxia.

Numerous studies support these findings. Narumiya et al. (2001) reported elevated plasma levels of MMP-2 in women with PE, which contrasts with our findings in late-onset PE patients, where we found MMP-2 to be downregulated [20]. This discrepancy may be explained by differences in the gestational age of onset and the specific placental subtypes of PE studied. A recent study on Early-onset PE, often linked to defective deep placentation, may involve compensatory MMP-2 upregulation as an attempt to restore trophoblast invasion, while late-onset PE, typically associated with metabolic stress, may show overall reduced MMP activity. Indeed, Timokhina et al. (2020) found similar gestational variations in placental MMP levels, underscoring the importance of differentiating between early- and late-onset PE [21].

In a study conducted by Arora et al., it was observed that placental samples from early-onset preeclamptic patients exhibited significantly upregulated mRNA and protein levels of TIMP-1 and TIMP-2 compared to normotensive, non-proteinuric controls, highlighting the inhibitory effect of TIMPs on MMP activity. [22].

Additionally, Ren et al (2018) emphasized the role of angiogenic factors in regulating MMP activity in preeclampsia. They suggested that impaired expression of VEGF and PlGF in PE could indirectly reduce MMP expression, leading to defective spiral artery remodeling [23]. Our findings of reduced MMP-2 and MMP-9 levels in preeclamptic placentas are consistent with this hypothesis, suggesting that impaired angiogenic signaling, possibly mediated by miR-30b, further contributes to MMP downregulation in PE.

Interestingly, while most studies, including those by [19], have focused on MMP-9 as the primary MMP dysregulated in PE, our study reveals a more nuanced picture involving both MMP-2 and MMP-9. The significant upregulation of TIMP-1 and TIMP-2 in both plasma and placental samples of PE patients adds further complexity to this regulatory network. By directly inhibiting MMP activity, TIMPs exacerbate the impaired invasion observed in PE, making them not just bystanders but active contributors to the disease pathology.

Clinical implications of miR-30b/CTH/MMP-TIMP axis in PE

The discovery that miR-30b modulates the CTH/MMP-TIMP axis in preeclampsia has significant clinical implications. Reduced CTH expression, as observed in our study, leads to a decrease in H₂S production, which correlates with impaired trophoblast invasion. Given the downregulation of MMPs and the upregulation of TIMPs in preeclamptic patients, targeting miR-30b may offer a therapeutic avenue for restoring normal placental function.

Several studies have proposed miRNA-based therapies for other diseases, such as cardiovascular disorders and cancers, where miR inhibition has shown promising results. For example, antagomirs (antisense miRNAs) have been successfully used to block specific miRNAs involved in disease progression. The use of miR-30b inhibitors in preeclampsia could restore CTH expression and H₂S levels, promoting trophoblast invasion and reducing the risk of placental insufficiency. Furthermore, the timing of intervention may play a critical role, as early inhibition of miR-30b could prevent the onset of severe placental pathology.

Additionally, our findings suggest that MMPs and TIMPs could serve as biomarkers for early detection of PE. Elevated TIMP-1 and TIMP-2 levels in the plasma of preeclamptic women, as observed in our study, may provide a non-invasive diagnostic marker for identifying women at risk of developing PE. The role of the miR-30/CTH/H_2_S pathway may contribute to the pathogenesis of PE.

## Conclusions

This study provides significant insights into the molecular mechanisms underlying PE by highlighting the critical role of miR-30b in regulating trophoblast invasion through modulation of the CTH/H₂S pathway and the MMPs and TIMPs axis. The role of the miR-30/CTH/H_2_S axis in placental development needs more elaboration. By employing both in vitro models and clinical samples, we confirmed that this dysregulation of MMPs and TIMPs in PE is closely tied to the pathological processes of placental ischemia, impaired spiral artery remodeling, and inadequate trophoblast function - the hallmarks of the disease. This study not only advances our understanding of the molecular drivers of PE but also lays the groundwork for the development of miR-30b-targeted therapies aimed at mitigating the effects of trophoblast dysfunction. Further research is warranted to explore the clinical applicability of miR-30b modulation and its potential in the early detection and treatment of PE. It is also required to explore a clear division in terms of early-onset patients and control subjects for future work.
